# *Echium acanthocarpum *hairy root cultures, a suitable system for polyunsaturated fatty acid studies and production

**DOI:** 10.1186/1472-6750-11-42

**Published:** 2011-04-27

**Authors:** Elena Cequier-Sánchez, Covadonga Rodríguez, Roberto Dorta-Guerra, Ángel G Ravelo, Rafael Zárate

**Affiliations:** 1 Bio-Organic University Institute AG González, University of La Laguna, Ave. Fco. Sánchez, 38206 La Laguna, Tenerife, Spain; 2 Canary Islands Cancer Research Institute (ICIC), La Candelaria University Hospital, Carr. El Rosario 145, 38010 Santa Cruz de Tenerife, Tenerife, Spain; 3 Animal Biology Dept., Biology Faculty, University of La Laguna, Ave. Fco. Sánchez, 38206 La Laguna, Tenerife, Spain; 4 Statistics and Computation Dept., Maths Faculty, University of La Laguna, Ave. Fco. Sánchez, 38206 La Laguna, Tenerife, Spain

## Abstract

**Background:**

The therapeutic and health promoting role of highly unsaturated fatty acids (HUFAs) from fish, *i.e. *eicosapentaenoic acid (EPA, 20:5n-3) and docosahexaenoic acid (DHA, 22:6n-3) are well known. These same benefits may however be shared by some of their precursors, the polyunsaturated fatty acids (PUFAs), such as stearidonic acid (SDA, 18:4 n-3). In order to obtain alternative sources for the large-scale production of PUFAs, new searches are being conducted focusing on higher plants oils which can contain these n-3 and n-6 C18 precursors, *i.e. *SDA and GLA (18:3n-6, γ-linolenic acid).

**Results:**

The establishment of the novel *Echium acanthocarpum *hairy root cultures represents a powerful tool in order to research the accumulation and metabolism of fatty acids (FAs) in a plant particularly rich in GLA and SDA. Furthermore, this study constitutes the first example of a *Boraginaceae *species hairy root induction and establishment for FA studies and production. The dominant PUFAs, 18:2n-6 (LA, linoleic acid) and 18:3n-6 (GLA), accounted for about 50% of total FAs obtained, while the n-3 PUFAs, 18:3n-3 (ALA, α-linolenic acid) and 18:4n-3 (SDA), represented approximately 5% of the total. Production of FAs did not parallel hairy root growth, and the optimal productivity was always associated with the highest biomass density during the culture period. Assuming a compromise between FA production and hairy root biomass, it was determined that sampling times 4 and 5 gave the most useful FA yields. Total lipid amounts were in general comparable between the different hairy root lines (29.75 and 60.95 mg/g DW), with the major lipid classes being triacylglycerols. The FAs were chiefly stored in the hairy roots with very minute amounts being released into the liquid nutrient medium.

**Conclusions:**

The novel results presented here show the utility and high potential of *E. acanthocarpum *hairy roots. They are capable of biosynthesizing and accumulating a large range of polyunsaturated FAs, including the target GLA and SDA fatty acids in appreciable quantities.

## Background

Stearidonic acid (SDA, 18:4n-3) and gammalinolenic acid (GLA, 18:3n-6) are scarce polyunsaturated fatty acids (PUFAs). They act as precursors for a range of physiologically essential highly unsaturated fatty acids (HUFAs), including eicosapentaenoic acid (EPA), docosahexaenoic acid (DHA) and arachidonic acid (ARA, 20:4n-6). EPA and DHA function as major animal nutrients, as well as being part of the cytoplasmatic membrane building elements that regulate membrane functions. Furthermore, EPA and ARA together with elongation products of SDA and GLA, are precursors of other important molecules including the eicosanoids [1]. In addition, HUFAs are of interest because of their important roles in human health and nutrition [2-7].

It is known that organisms able to synthesize HUFAs follow two different pathways; the more common aerobic pathway utilizes desaturase and elongase enzymes, while the anaerobic pathway is catalyzed by polyketide synthases [8]. The former consists of consecutive elongation and desaturation cycles of the carbon chain. According to the order in which desaturation and/or elongation proceed, several pathway variations exist.

There exists another unusual pathway described in mammals and fish, *i.e. *the Sprecher´s pathway. This route is characterized by a lack of desaturation reaction at Δ4-position, but successive Δ5 and Δ6-desaturations of α-linolenic acid (ALA, 18:3n-3), generating a six double bond C24 intermediate which is finally shortened by peroxisomal β-oxidation eventually forming DHA [9].

Mammals, including humans, are poor convertors of the precursors linoleic acid (LA; 18:2n-6) and ALA into HUFAs; therefore, HUFAs must be taken up directly as components of the diet [10]. The current main dietary source of n-3 HUFAs is fish and other seafood. The FAs are initially produced by a plethora of marine microorganisms, which proceed through the food chain and end up in fish [11]. Unfortunately, the increased demand for fish and fish oils has led to depletion of fish stocks worldwide [12]. Because of this, fish farming has developed into a highly productive and efficient industry [13]. Thus, aquaculture and particularly fish production might be the future source of n-3 HUFAs, although it also depends on extractive fishing for producing fish fodder.

In order to obtain a more suitable source for the large-scale production of PUFAs, searches for new sources of these compounds have been conducted [14]. One potential solution is to seek alternative sources of higher plant oils which contain the health promoting C18 precursors, SDA and GLA. It is known however that very few plant species, mainly *Boraginaceae, Onagraceae, Saxifragaceae *and *Scrophulariaceae *families [15] have the biosynthetic capacity to produce and accumulate Δ6-desaturated FAs predominantly in the form of the n-6 GLA, and in the case of the n-3 series, SDA. The latter in particular is a very rare FA found in few higher plant oils offering greater interest due to its known medicinal properties. Interestingly, SDA has not been qualified as equivalent to EPA and DHA as an essential FA, unlike EPA and DHA, although it has been shown to possess similar beneficial health properties as the longer chain n-3 HUFAs [16-18].

Consequently, we focused our attention on the *Echium *genus (*Boraginaceae*) as a potential source of higher plant oil. The *Echium *genus comprises 60 species distributed in various continents, in the Canary Islands it has a large biodiversity with 23 endemic species having been described [19]. They constitute one of the largest plant sources of SDA and GLA [20,21], as well as offering an attractive n-3/n-6 balance.

Other reported benefits of a SDA and/or GLA rich FA profile include their use to control the production of proinflammatory eicosanoids derived from ARA [18,22,23], and to avoid excessive fatty tissue deposition [24-27].

It is well known that secondary metabolite production in plants is strongly influenced by meteorological factors [28]; therefore, *in vitro *plant cultures, especially, the highly differentiated hairy root cultures, obtained after guided infection with *Agrobacterium rhizogenes*, offer an attractive alternative for the stable production and study of natural products including PUFAs [29-31].

To the best of our knowledge, the present study is the first example of the establishment of hairy roots for the study and production of PUFAs, and the first account of a *Boraginaceae *species hairy root culture. Here, a detailed study on the establishment of *E. acanthocarpum *hairy root cultures and their ability to synthesize and accumulate PUFAs is reported, proving to be a suitable system for the production of n-3 and n-6 healthy PUFAs.

## Results

### Characterization of hairy root cultures and growth

The two well-established transformed root cultures (*HR E1.5 *and *HR E1.16*) were clones showing a medium growth rate and high stability with regards to their FAs production. Morphologically the roots of line *HR E1.5 *were much thicker, shorter and less branched than those of *HR E1.16*. Both, slight callus and hyper-hydrated tissue formation were occasionally observed in the *HR E1.5 *hairy roots (Additional file 1, Figure S1a, b, c,). Despite having the insertion of the *npt-II *gene, which provides resistance to kanamycin (Kn), as determined by PCR (Additional file 1, Figure S1d) only *HR E1.16 *was able to grow in the presence of the antibiotic; regardless of this, the two hairy root lines were investigated.

**Figure 1:**
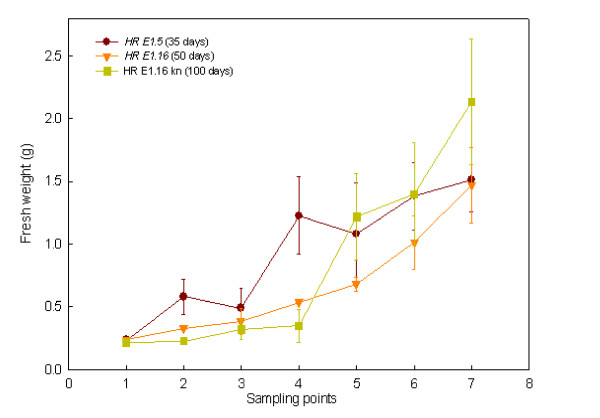
**Growth of the two hairy root lines**. Growth of hairy roots along each experimental period. Each value is the mean ± standard deviation of three replicates expressed in grams. Kn = kanamycin. The different sampling point days are specified in the Method section.

The growth and FA production were monitored for either 35, 50 or 100 days. Line *HR E1.5 *grew faster and reached its maximum fresh weight (FW) after 30 days (1.51 g), although statistical differences were not detected from day 20 onwards (Figure 1). On the other hand, *HR E1.16 *line grown in the presence or absence of kanamycin, generated similar biomasses to *HR E1.5 *(2.14 and 1.47 g FW, respectively), but grew for longer time periods *i.e. *100 and 50 days respectively (Figure 1).

As expected, statistical analysis revealed significant differences between FW values obtained at different sampling points in each culture, describing a typical growth profile *i.e. *latent phase, exponential phase and stationary phase (Figure 1). Nonetheless, no significant differences were observed when comparing the maximum values of FW of the three cultures.

### Fatty acid profiles. Time course of lipid and PUFA production

Since very little is known about lipid and FA production by transformed hairy root cultures, a preliminary study of the whole lipid composition was conducted. Regarding total lipid amounts (TL), no statistical differences were detected among triplicates of different samplings within the same hairy root line. Additionally, TL extracted from the two hairy root lines (*HR E1.5 *and *HR E1.16*) gave comparable results (Table 1; Additional file 1, Tables S1, S2, S3, S4, S5), *HR E1.5 *gave values between 31.38 and 60.95 mg TL/g DW, while *HR E1.16 *gave values between 29.75 and 45.29 mg TL/g DW. The major lipid classes were triacylglycerols, 22.02% and 13.77% for *HR E1.5 *and *HRE1.16*, respectively, and sterols ester (19.88% in *HR E1.5 *and 14.70% in *HRE1.16*) (*data not shown *[32]).

**Table 1:** Sampling points 4 and 5

*Sampling point 4*	*HR E1.5 (20 days)*	*HR E1.16 (28 days)*	*HR E1.16 with Kn (56 days)*
*Total Lipid content (mg/g DW)*	*33.41 ± 3.66*	*41.55 ± 4.64*	*27.13 ± 3.41*
*14:0*	*0.22 ± 0.02*	*0.43 ± 0.05*	*0.43 ± 0.04*
*16:0*	*26.53 ± 1.73*	*23.91 ± 1.49*	*22.56 ± 0.67*
*18:0*	*2.92 ± 0.30*	*4.10 ± 0.28*	*4.02 ± 0.27*
*18:1n-9*	*4.56 ± 0.92*	*5.53 ± 1.00*	*5.61 ± 0.72*
*18:1 n-7*	*1.53 ± 0.05*	*1.19 ± 0.09*	*1.03 ± 0.17*
*18:2n-6 (LA)*	*36.82 ± 0.99*	*41.39 ± 0.95*	*40.16 ± 1.55*
*18:3n-6 (GLA)*	*13.88 ± 0.78*	*10.48 ± 0.27*	*10.55 ± 0.53*
*18:3n-3 (ALA)*	*4.24 ± 0.20*	*3.54 ± 0.25*	*4.61 ± 1.75*
*18:4n-3 (SDA)*	*1.26 ± 0.45*	*0.68 ± 0.00*	*0.84 ± 0.30*
*20:0*	*0.29 ± 0.06*	*0.40 ± 0.08*	*0.37 ± 0.05*
*22:0*	*2.45 ± 0.31*	*2.58 ± 0.36*	*2.50 ± 0.32*
*24:0*	*1.94 ± 0.49*	*2.49 ± 0.28*	*2.72 ± 0.77*
*Unknown*	*2.36 ± 0.35*	*2.64 ± 0.08*	*2.65 ± 0.15*
*Fatty acids (% of total lipid content)*	*22.17 ± 4.23*	*23.96 ± 4.02*	*18.12 ± 2.43*
GLA and SDA	*15.14 ± 1.23*	*11.16 ± 0.27*	*11.39 ± 0.83*
Saturated fatty acids	*34.36 ± 1.67*	*33.90 ± 1.92*	*32.61 ± 0.99*
Monoene fatty acids	*7.07 ± 0.73*	*7.37 ± 0.82*	*7.17 ± 0.87*
n-9	*5.33 ± 0.66*	*6.04 ± 1.11*	*6.14 ± 0.79*
n-6	*50.70 ± 1.66*	*51.87 ± 0.97*	*50.72 ± 1.06*
n-3	*5.50 ± 0.53*	*4.22 ± 0.25*	*5.45 ± 2.05*
n-3/n-6	*0.11 ± 0.01*	*0.08 ± 0.00*	*0.11 ± 0.04*
*n-6 Δ6-Desaturation Index*	*0.27 ± 0.01*	*0.20 ± 0.01*	*0.21 ± 0.01*
*n-3 Δ6-Desaturation Index*	*0.23 ± 0.06*	*0.16 ± 0.01*	*0.15 ± 0.00*
DBI	*1.40 ± 0.06*	*1.35 ± 0.03*	*1.36 ± 0.04*

***Sampling point 5***	*HR E1.5* *(25 days)*	*HR E1.16* *(35 days)*	*HR E1.16 with Kn* *(70 days)*

*Total Lipid content (mg/g DW)*	*35.00 ± 5.40*	*29.75 ± 1.63*	*34.34 ± 5.25*
*14:0*	*0.23 ± 0.02*	*0.27 ± 0.06*	*0.38 ± 0.08*
*16:0*	*25.04 ± 1.14*	*23.63 ± 0.45*	*25.23 ± 0.27*
*18:0*	*2.55 ± 0.21*	*4.06 ± 0.21*	*3.93 ± 0.18*
*18:1n-9*	*6.77 ± 1.10*	*6.12 ± 0.44*	*4.01 ± 0.24*
*18:1 n-7*	*1.55 ± 0.16*	*0.86 ± 0.23*	*0.99 ± 005*
*18:2n-6 (LA)*	*34.55 ± 1.97*	*42.89 ± 1.31*	*38.05 ± 0.55*
*18:3n-6 (GLA)*	*11.77 ± 1.63*	*10.05 ± 0.40*	*11.95 ± 0.31*
*18:3n-3 (ALA)*	*4.22 ± 0.34*	*3.69 ± 0.23*	*5.12 ± 0.26*
*18:4n-3 (SDA)*	*0.88 ± 0.21*	*0.58 ± 0.03*	*1.09 ± 0.22*
*20:0*	*0.27 ± 0.04*	*0.42 ± 0.02*	*0.46 ± 0.02*
*22:0*	*2.51 ± 0.35*	*2.59 ± 0.19*	*2.76 ± 0.09*
*24:0*	*2.08 ± 0.60*	*2.25 ± 0.45*	*2.69 ± 0.24*
*Unknown*	*6.08 ± 2.68*	*2.03 ± 0.44*	*2.87 ± 0.17*
*Fatty acids (% of total lipid content)*	*24.53 ± 4.16*	*26.86 ± 3.29*	*27.27 ± 9.10*
GLA and SDA	*12.65 ± 1.84*	*10.63 ± 0.43*	*13.04 ± 0.53*
Saturated fatty acids	*32.68 ± 2.21*	*33.22 ± 1.21*	*35.45 ± 0.07*
Monoene fatty acids	*9.54 ± 1,29*	*7.53 ± 0.55*	*5.48 ± 0.15*
n-9	*7.59 ± 1.15*	*6.66 ± 0.50*	*4.38 ± 0.08*
n-6	*46.32 ± 3.60*	*52.94 ± 1.67*	*50.00 ± 0.47*
n-3	*5.09 ± 0.53*	*4.28 ± 0.23*	*6.21 ± 0.41*
n-3/n-6	*0.11 ± 0.01*	*0.08 ± 0.00*	*0.12 ± 0.01*
*n-6 Δ6-Desaturation Index*	*0.25 ± 0.02*	*0.19 ± 0.00*	*0.24 ± 0.01*
*n-3 Δ6-Desaturation Index*	*0.17 ± 0.02*	*0.14 ± 0.01*	*0.17 ± 0.03*
DBI	*1.30 ± 0.08*	*1.37 ± 0.04*	*1.37 ± 0.00*

Total lipid content and general fatty acid profiles (%) of two cell lines of *Echium acanthocarpum *hairy roots at sampling points 4 and 5 cultured in B5 nutrient liquid medium at 25°C. n-6 and n-3 Δ6-Desaturation Indexes were calculated as 18:3n-6/(18:3n-6+18:2n-6) and 18:4n-3/(18:4n-3+18:3n-3), respectively. Double Bond Index was calculated as [(% 18:1) +2*(% 18:2) +3*(% 18:3) +4*(18:4)]/100. Values are presented as the average of three replicates (data of samplings 4&5 are presented here, all remaining data sets are given in the Additional file 1. (The different sampling point days are also specified in the Method section).

**Figure 2:**
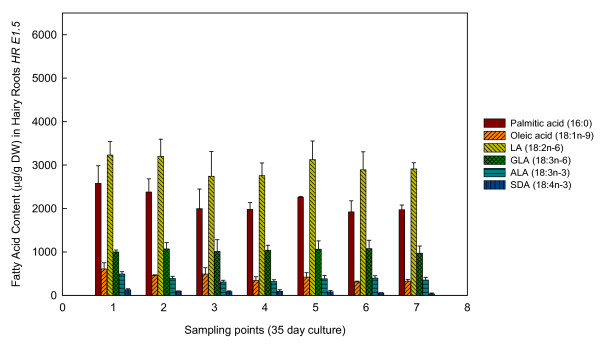
**Fatty acid profiles of line *HR E1.5***. Amounts of different fatty acids obtained from *HR E1.5 Echium acanthocarpum *hairy roots cultures during a 35-day period. Each value is the mean ± standard deviation of three replicates expressed as micrograms per gram of DW. The different sampling point days are specified in the Method section.

**Figure 3:**
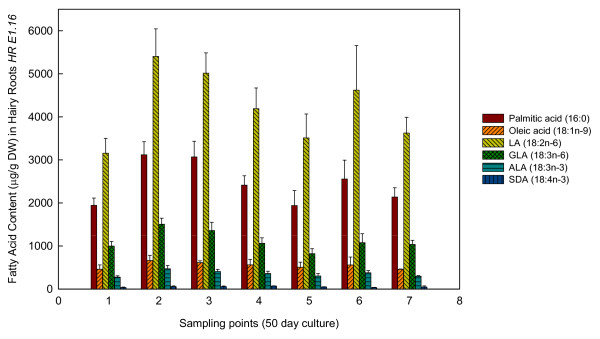
**Fatty acid profiles of line *HR E.16***. Amounts of different fatty acids obtained *HR E1.16 Echium acanthocarpum *hairy roots cultured without kanamycin presence during a 50-day period. Each value is the mean ± standard deviation of three replicates expressed as micrograms per gram of DW. The different sampling point days are specified in the Method section.

**Figure 4:**
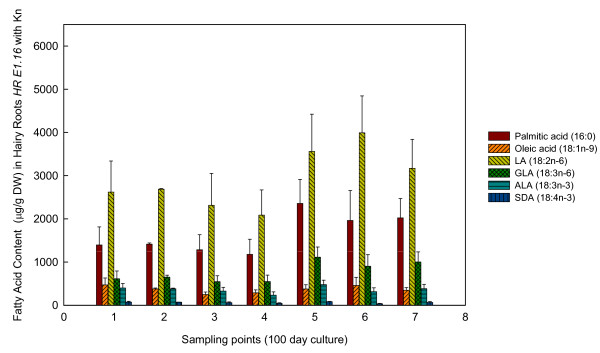
**Fatty acid profiles of line *HR E1.16 *with kanamycin**. Amounts of different fatty acids obtained from *HR E1.16 Echium acanthocarpum *hairy roots cultured in kanamycin presence during a 100-day period. Each value is the mean ± standard deviation of three replicates expressed as micrograms per gram of DW. The different sampling point days are specified in the Method section.

Concerning FAs production in *E. acanthocarpum *hairy roots, these compounds were chiefly stored and present in the hairy roots, and very minute amounts were released into the liquid nutrient medium. Furthermore, hairy roots were able to accumulate important amounts of FAs, in particular the saturated palmitic acid (16:0) and stearic acid (18:0), together with lower amounts of 20:0, 22:0 and 24:0 (Figures 2, 3 and 4, Table 1). Unsaturated FAs including oleic acid (18:1n-9), 18:1n-7, 18:2n-6, 18:3n-3, as well as the Δ6-desaturated target FAs, γ-linolenic acid (18:3n-6, GLA) and stearidonic acid (18:4n-3, SDA), were also observed (Figures 2, 3 and 4, Table 1).

In both hairy root lines, the dominant PUFAs were 18:2n-6 and 18:3n-6 in descending order, which accounted for about 50% of total FAs (Figures 2, 3 and 4, Table 1). The n-3 PUFAs, 18:3n-3 and 18:4n-3, appeared in lower amounts, representing approximately 5% of total FAs. Other FAs were also identified and quantified. The saturated FAs (16:0, 18:0, 20:0 and 24:0) were extracted in relatively large amounts, *i.e. *32-38% of total FAs; whereas, monoenes only represented about 5-9% of total FAs (Table 1; Additional file 1, Tables S1, S2, S3, S4, S5).

### Effect of hairy root cell line and culture time on fatty acid production - *HR E1.5*

The final FA profiles obtained from *HR E1.5 *hairy roots, after 35 days of culture, were very similar (Figure 2, Table 1; Additional file 1, Tables S1, S2, S3, S4, S5). Statistical analyses over the time-course of the experiment showed temporal changes in the amounts of individual FA present in *HR E1.5*. The percentage of 18:0 for example was slightly higher in sampling point 1 (5 d. of culture) compared to other time points while 18:1n-9 was slightly larger in sampling point 5 (25 d. of culture) (Figure 2, Table 1; Additional file 1, Tables S1, S2, S3, S4, S5). The percentage of 18:2n-6 was found to be significantly higher in sampling points 6 and 7 (38.13 and 38.75% of total FAs) (Additional file 1, Tables S3, S4, S5). On the other hand, several saturated FAs such as, 14:0, 20:0, 22:0 and 24:0, reached different percentages at each sampling, between 0.15 to 0.83% for 14:0; 0.19 to 0.41% for 20:0; 2.06 to 2.81% for 22:0, and 1.40 to 2.08% for 24:0. (Table 1; Additional file 1, Tables S1, S2, S3, S4, S5).

The n-6 Δ6-Desaturation Index [GLA/(LA+GLA)] which provides information on the proportions of n-6 FAs within the samples, was also determined for each hairy root line. For *HR E1.5*, the ratio was slightly lower, 0.24 for sampling point 1 than for the rest of sampling points (0.25-0.27) but no statistical difference was found. Likewise, the ratios n-3 Δ6-Desaturation Index [SDA/(ALA+SDA)], which provide information on the proportions of unsaturated n-3 FAs within the samples, and the DBI (Double Bond Index), which establishes the richness of unsaturated FAs within the samples, were also determined for each hairy root line. Again, no statistical differences were observed. (Table 1, Additional file 1, Tables S4-S5).

In this report, FA production studies were carried out taking into consideration both the percentage of each FA, as well as the absolute amounts of these. In relation to absolute values, 18:2n-6 (LA) was the most abundant FA, with over 3 mg/g DW at sampling points 1 and 2, followed by 16:0 with 2.57 mg/g DW at the same sampling points, and 18:3n-6 with 1.06 mg/g DW at sampling point 5 (Figure 2). The n-3 FAs were generally detected in lower amounts 18:3n-3 with 0.38 mg/g DW, and 18:4 n-3 with 0.08 mg/g DW at sampling point 5 (Figure 2).

### Effect of hairy root cell line and culture time on fatty acid production - *HR E1.16*

Similar results were observed for the *HR E1.16 *hairy root cultures, the FA profiles at the end of the experimental period (50 or 100 days of culture), being only marginally different from those observed from *HR E1.5*.

Again some temporal changes were noted. For example, in cultures without added Kn, 18:3n-6 (GLA) gave peak percentages at sampling points 1-3 and 7 with significantly lower percentages being observed at points 4 and 5. This was also reflected in a statistically lower n-6 Δ6-Desaturation Index (p ≤ 0.05) at these same time points (values of 0.19-0.20).

Apart from this, n-3 PUFAs including ALA (18:3n-3) and SDA (18:4n-3) levels were clearly lower than n-6 PUFAs, with the highest ALA percentages occurring at sampling point 4 (3.69% of total FAs), although no statistical differences were detected throughout the experiment. In a similar fashion, SDA (18:4n-3) levels were slightly elevated at sampling point 4 (0.58% of total FAs), but no significant differences were detected, the mean values being 0.45% of total FAs throughout the experiment.

The n-3 Δ6-Desaturation Index or [SDA/(ALA+SDA)] ratio, was lower at sampling 6, with its maximum values being achieved at sampling 4 (0.16). Only a few statistical differences were detected in saturated FAs. FAs 22:0 and 24:0 were statistically lower at sampling point 7 (Additional file 1, Table S5) while the monoenes 18:1n-7 and 18:1 n-9, and the saturated FAs 16:0, 18:0, 20:0 were statistically similar at all sampling points. Similarly, no statistical differences were detected in the DBI Index throughout samplings 2-6 (values 1.35-1.37). In reference to absolute FA amounts, 18:2n-6 was again the most abundant, yielding a maximum of 5.40 mg/g DW (sampling point 2) and a minimum of 3.52 mg/g DW at sampling point 1. This was followed by 16:0 (3.91 mg/g DW at sampling 2 and 1.91 mg/g DW, at sampling point 5); and 18:3n-6, which displayed a range between 0.82 mg/g DW (sampling point 5) and 1.50 mg/g DW (sampling points 2). As expected, the values for n-3 FAs were lower, with maximum quantities achieved at sampling point 2 for 18:3n-3 (0.47 mg/g DW), and sampling point 4 for 18:4n-3 (0.07 mg/g DW) (Figure 3).

When the same hairy root line was cultured in the presence of kanamycin, the FA profile was characterized by a particular abundance of 18:2n-6 (38-40% of total FAs), and 16:0 (20-25% of total FAs) showing statistical differences at samplings points 5-7 (p ≤ 0.05). GLA was also abundant in the pool, and accounted for up to 12.02% of total FAs (sampling point 7). Similarly to the other two hairy root cultures, the percentages of 18:3n-3 and 18:4n-3 were lower than their n-6 cousins (5.12%, and 1.09% of total FAs respectively, sampling 5) (Table 1). The maximum value for the monoene 18:1n-9 was observed at sampling 1 and 2 (8.26 and 7.2%, respectively) while the level of the other monoene 18:1n-7, as well as the saturated FAs 14:0, 18:0, 20:0 and 22:0 were constant throughout the experiments (Additional file 1, Tables S1, S2, S3, S4, S5).

**Figure 5:**
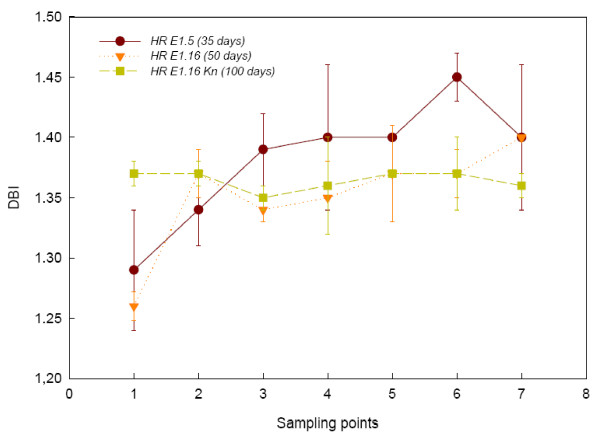
**Double Bond Index**. Double Bond Indexes (DBI) of each culture were calculated as [(% 18:1) +2*(% 18:2) +3*(% 18:3) +4*(18:4)]/100. Each value is the mean ± standard deviation of three replicates. The different sampling point days are specified in the Method section.

According to the preceding data, the ratios [GLA/(LA+GLA)] and [SDA/(ALA+SDA)] reached their maximum values at sampling point 5 (0.24 and 0.17, respectively) (p ≤ 0.05), indicating a maximum Δ6-desaturation activity. In reference to DBI (1.37), no statistical differences were detected throughout the course of the experiments (Figure 5).

The absolute amounts of FAs generated from the *HR E1.16 *line with kanamycin maintained the same order as the previous experiments with 18:2n-6 (LA) being the most abundant, reaching 3.55 mg/g DW at sampling point 5, followed by 16:0 (2.35 mg/g DW at sampling 7), and 18:3n-6 (1.11 mg/g DW at sampling point 5) (Figure 4). As expected the n-3 FAs were observed in lower amounts than the n-6 FAs, reaching maximum values of 0.47 mg/g DW for 18:3n-3 and 0.10 mg/g DW for 18:4n-3 at sampling point 5 (Figure 4).

**Figure 6:**
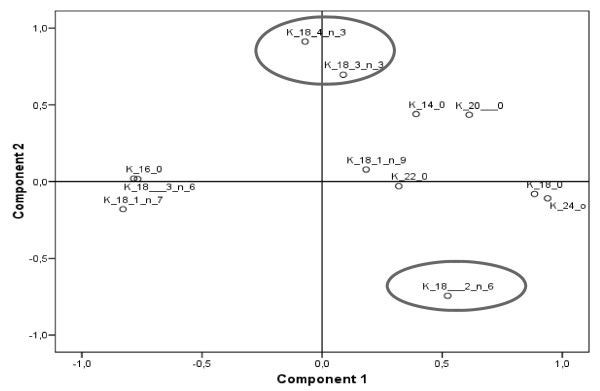
**Factor loading plots**. Factor loadings plots for the percentages of fatty acids.

Principal component analysis (PCA) was also conducted in order to reduce the dimensionality of the FA variables, and to visualize this problem from a two-dimensional point of view. The percentage of each FA variable was considered generating two principal components, PC1 and PC2, with a cumulative explained variance of 54.44%. PC1 component (cumulative explained variance = 36.29%) was positively correlated with certain saturated FAs (14:0, 18:0, 20:0, 22:0, 24:0) and strongly negatively correlated with 16:0, 18:1n-7 and 18:3n-6 (Figure 6). Accordingly, the PC1 component correlated with the level of saturation, indicating that a higher value would correspond to more saturated FA profile. Furthermore, PC2 (cumulative explained variance = 18.14%), was positively correlated with 18:3n-3 and 18:4n-3, and negatively related with 18:2n-6 (Figure 6). As a result, the new variable PC2 was clearly associated with FAs of the n-3 series, and in general, with polyunsaturated FAs.

PCA analysis of absolute amounts of FAs showed similar results, *i.e. *the same correlations than in the first analysis. This is probably due to the fact that no significant differences were detected for TL (p ≥ 0.05), fresh weight (p ≥ 0.05) (Figure 1) or for lipid classes (*data not shown*).

**Figure 7:**
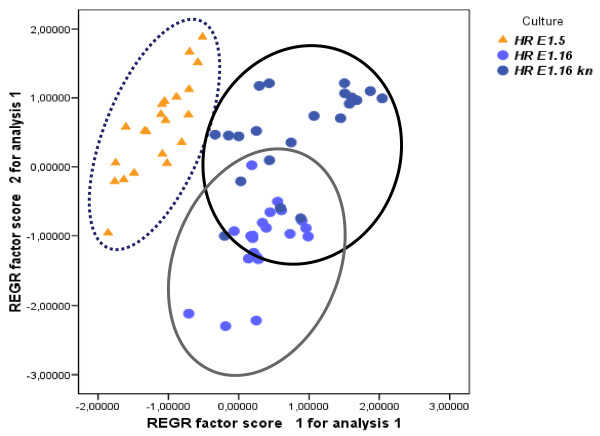
**Production of fatty acids depending on type of culture**. Plot of PC1 and PC2 factor scores categorized by type of culture.

**Figure 8:**
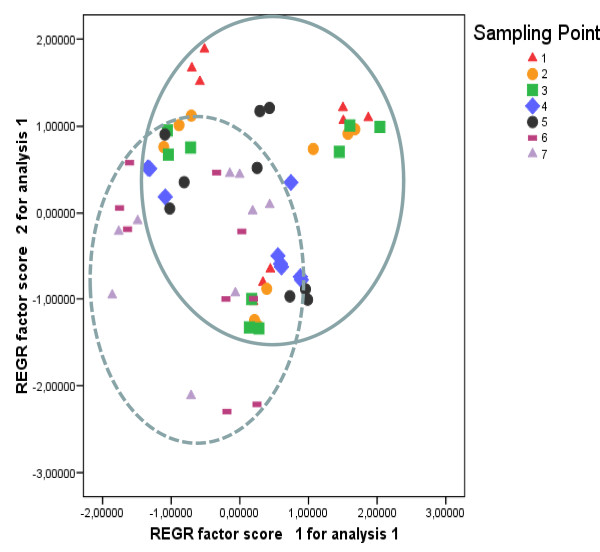
**Production of fatty acids depending on time of culture**. Plot of PC1 and PC2 factor scores categorized by sampling points.

A two-way ANOVA analysis for both components, PC1 and PC2 was also conducted. This study showed that the effects of the culture, sampling point, and the interaction of the culture and sampling point, were significant on each component (Figures 7 and 8, Additional file 1, Table S6), with a particular emphasis on PC2, which represents an enrichment of n-3 FAs in the extracted oil. According to the statistical data, the average of the second component PC2 categorized by sampling points was found to be strongly intensified between sampling points 1-5 for the three cultures (Additional file 2, Figure S1). These data were correlated with the [SDA/(ALA+SDA)] ratio at these days (0.17-0.21 for *HR E1.5*, 0.10-0.14 for *HR E1.16 *without kanamycin and 0.15-0.17 for *HR E1.16 *with kanamycin). Furthermore, the averages of the PC2 component categorized by the combination of the kind of culture and by the sampling points, appeared to be higher in *HR E1.5 *and *HR E1.16 *with added kanamycin (Additional file 2, Figure S2), and correlated with the ratios [SDA/(ALA+SDA)] and [GLA/(LA+GLA)] recorded at these points and in these cultures (*i.e. *Table 1). In general, hairy root line *HR E1.5 *accumulated more n-3 desaturated FAs, and less saturated ones, reflected by the PC1 component (Additional file 2, Figure S3). In addition, *HR E.16 *showed similar levels of n-3 desaturated FAs when these hairy roots were cultured in the presence of kanamycin. Nonetheless, the levels of saturated FAs were always higher compared to *HR E1.5 *line (Additional file 2, Figure S3). Therefore, the n-6 and n-3 Δ6-Desaturation index were significantly higher in *HR E1.5 *and in *HR E.16 *cultured with kanamycin (p = 0.511 and p = 0.788, respectively) (Table 1; Additional file 1, Tables S1, S2, S3, S4, S5).

## Discussion

It has been shown that the establishment of the novel *E. acanthocarpum *hairy root cultures represents a powerful tool to aid research regarding the accumulation and metabolism of FAs. Moreover, this is of particular interest given that these plants are an unusual rich source of GLA and SDA. The attractiveness of using transgenic plants or cultures as green-factories for the synthesis of high value product has been documented [33-37]. The main objective of this work was to assess the utility of this new system with regards to the study of FA metabolism and production. We focused on the production of PUFAs due to their known health benefits with a particular emphasis on omega-3 stearidonic acid (SDA), a precursor of valued HUFAs, such as EPA and DHA, and n-6 GLA, a known modulator of eicosanoids derived from ARA. Furthermore, SDA and GLA are particularly valuable in view of the fact that the initial Δ6-desaturation of their precursors ALA and LA, respectively appears to be the rate-limiting step of the entire pathway [38], and that very few plant species are able to further metabolise ALA into SDA.

Although roots are generally considered structural organs providing physical support to maintain the stability of the plant, and appear rich in phospholipids and sterol esters [39], they are also potent biochemical factories, able to biosynthesize and often accumulate a myriad of bioactive secondary metabolites [33-35], including FAs as first reported here.

Hairy roots induced by *Agrobacterium rhizogenes *transformation are widely used for the study of metabolic regulation and large-scale metabolic production because, biosynthesis in hairy roots mimics or even improves that of intact roots in the original plants, or intact plants [35,37]. This study constitutes the first report on the induction and establishment of a *Boraginaceae *species hairy root culture for the study of FA production. *Echium acanthocarpum*, together with the other twenty-two Canary Islands endemic *Echium *species are known as FAs containing plants, the majority of which are located in its seeds and leaves [20,21]. Nevertheless, the FAs content has never been investigated in intact *E. acanthocarpum *plant roots. In this study, our *E. acanthocarpum *hairy root system was found capable of growing in an stable and amenable manner, and more importantly, it was able to produce and accumulate a consistent profile of FAs including the PUFAs 18:2n-6 (LA) and 18:3n-6 (GLA).

In order to better characterize the PUFAs production efficiency of the *E. acanthocarpum *hairy roots, two cell lines (*HR E1.5 *and *HR E1.16*) were cultured under similar conditions. Independently of the hairy root line, the most abundant FA was 18:2n-6 (LA), whose values were between 33.57-43.54%. These values are in agreement with previous reports using intact roots of *E. asperrimum *(30.05% of LA), and other *Boraginaceae *roots with 26.1% [40]. The presence of 18:2n-6 in the seeds and leaves of *E. acanthocarpum *has also been reported but in lower amounts (Table 2)[20,21].

**Table 2:** Comparison between different fatty acids profiles

*Fatty acids (%)*	*HR E 1.5 (sampling point 4)*	***E. asperrimum roots ***[40]	***E. acanthocarpum seeds ***[20]	***E. acanthocarpum leaves***[21]	**Transgenic *Arabidopsis seeds***[45]
*16:0 and 16:1-7*	*26.53*	*21.33*	*6.13*	*12.81*	*8.7*
*18:0*	*2.92*	*2.99*	*3.90*	*3.23*	*2.9*
*18:1n-9*	*4.56*	*5.78*	*11.49*	*7.62*	*14.4*
*18:1n-7*	*1.53*	*0.46*	-	-	-
*18:2n-6 (LA)*	*36.82*	*30.05*	*19.26*	*14.30*	*27.3*
*18:3n-6 (GLA)*	*13.88*	*8.22*	*24.51*	*1.88*	*8.5*
*18:3n-3 (ALA)*	*4.24*	*9.98*	*24.23*	*9.32*	*13.7*
*18:4n-3 (SDA)*	*1.26*	*3.40*	*7.45*	*1.45*	*2.6*
*C20*	*0.29*	*1.49*	*0.82*	*2.21*	*14.1*
*C22*	*2.45*	*2.40*	*0.21*	*1.34*	-
*C24*	*2.08*	*0.32*	*0.15*	*1.84*	-
*Fatty acids (% of weight)*	*0.84*	*0.53*	*15.07*	*1.68*	*1.40*
GLA and SDA	*15.14*	*11.62*	*31.96*	*3.33*	*11.1*
Saturated fatty acids	*34.36*	*28.73*	*9.93*	*21.43*	*13.3*
Monoene fatty acids	*7.07*	*6.36*	*12.76*	*7.62*	*26.8*
n-9	*5.33*	*5.9*	*12.40*	*7.62*	*14.4*
n-6	*50.70*	*38.27*	*43.77*	*16.18*	*35.8*
n-3	*5.50*	*13.38*	*31.68*	*10.77*	*16.3*
n-3/n-6	*0.11*	*0.35*	*0.72*	*0.66*	*0.45*
*n-6 Δ6-Desaturation Index*	*0.27*	*0.21*	*0.55*	*0.11*	*0.23*
*n-3 Δ6-Desaturation Index*	*0.23*	*0.25*	*0.76*	*0.13*	*0.15*

Comparison of fatty acids profiles (%) of *HR E1.5 *hairy roots with *Echium asperrimum *roots, *E. acanthocarpum *seeds and leaves, and transgenic *Arabidopsis *seeds expressing the *E. plantagineum *Δ6-desaturase gene. n-6 and n-3 Δ6-Desaturation Indexes were calculated as 18:3n-6/(18:3n-6+18:2n-6) and 18:4n-3/(18:4n-3+18:3n-3) respectively. Double Bond Indexes (DBI) were calculated as [(% 18:1) +2*(% 18:2) +3*(% 18:3) +4*(18:4)]/100. Numbers within brackets indicate reference number.

Furthermore, attractive amounts of n-3 unsaturated PUFAs, *i.e. *ALA and SDA, have also been reported in this study, the levels of SDA being specially interesting as it can serve as a dietary precursor of the valued eicosapentaenoic acid (20:5n-3) [18]. It was noted that the increase in total amount of PUFAs did not parallel to hairy root growth. Maximum production appeared at the early stages or active growth phase of each of the three cultures studied. This might be explained by the increasing amount of actively dividing meristematic root cells and, subsequently by membrane cytoplasmatic formation [30]. However, the optimal PUFA productivity in this hairy root system was always associated with the highest biomass density during the culture period; therefore, assuming a compromise between FA production and hairy root biomass, it was determined that sampling times 4-5 gave the most beneficial FA yields (Figures 2, 3 and 4).

Furthermore, it is known that seeds are, by nature, specialized storage organs. For example it has been reported that *E. acanthocarpum *seeds are characterized by 15.07% of FAs (Table 2) [20], 70-80% of which are triacylglycerol (TAG), the fraction were the FA pool is mostly present. In addition, it has also been reported that the chloroplast membrane is also able to accumulate a high percentage of lipids, known to accumulate up to 75% of total FAs indicating the importance of the aerial chlorophyllic organs in FA production [21,41]. Taking this into account, species of plants subjected to a higher solar irradiation would have smaller chloroplast membranes in their leaf cells and subsequently a lower percentage of FA in their leaf oil. Accordingly, leaf oil content was higher in European *Echium *species (mean value 2.72% FA/weight) than in Macaronesian *Echium *plants (mean value 1.52%). For the case of *E. acanthocarpum*, its leaf oil value was 1.84%, and our hairy root system yielded 0.84% (Table 2). Here the *E. acanthocarpum *hairy roots were able to accumulate FAs although cultured in the dark which would also hinder the formation of chlorophyllic cells, thus not having a full FA production potential of abundant chloroplast membranes. The data of *E. acanthocarpum *hairy roots FAs appear to be in agreement with the FAs content reported in *E. asperrimum *plant roots although showing lower values in the intact roots of this species (0.54%, Table 2) [40].

Although it is generally accepted that seed represents the best source of GLA [42,43], attractive amounts of FAs have been reported in other plant organs such as leaves [20,40,44]. The Δ6-desaturated 18:3n-6 was particularly abundant in the hairy roots, especially in *HR E1.5*, with 13.88%, after only 20 days of culture (sampling point 4). This is a significant percentage, especially when compared to other natural sources of this FA, such as the *Ribes sp*. seeds (19%) [43] or the *Echium sp*. seeds (*i.e. *26.31% of total FA in *E. callithyrsum*) [20]. The amount of GLA, as a percentage of the total FA content in *E. acanthocarpum *seeds, was higher than in our hairy roots, correlating with the n-6 Δ6-Desaturation Index (Table 2). Nonetheless, the high relative percentage of GLA accumulated by *HR E1.5 *is comparable to the values reported for *E. acanthocarpum *leaves [21], *E. asperrimum *roots [40], and even *Arabidopsis *seeds overexpressing a *E. plantagineum *Δ6-desaturase gene [45] (Table 2).

Although the n-3 PUFAs were clearly in lower proportions than the n-6 series FAs, ALA and SDA constituted a significant proportion of the total percentage of FA present in both the *HRE1.5 *and *HR E1.16 *in kanamycin presence (4-6% and 0.76-1.33% of total FA, respectively). Metabolically, the presence of low levels of ALA in these hairy roots should lead to the reduced production of SDA, compared to the larger production of GLA, since the levels of the n-3 precursor of the same Δ6-desaturase enzyme was not as abundant as the n-6 precursor. This could be supported by the n-3 Δ6-Desaturation Index, which was comparable to the examples reviewed in Table 2, except for seeds.

In order to reduce the dimensionality of this multivariate problem and investigate a possible correlation between FA profiles, the kind of hairy root line and the different sampling points, a PCA analysis with the obtained FA data was conducted. Two components were extracted with a cumulative explained variance of 54.44%. The PC2 was positively correlated with the n-3 FA variables (Figure 6).

Regarding the FA profiles, the majority of the results were similar, showing no statistical differences. The *HR E1.16 *cultured without added kanamycin did however show lower n-3 FA amounts and lower Δ6-desaturation activity (Figure 7 and Additional file 2, *Figure S*2). This could be attributed to the different phenotypes of the roots and the selection pressured ejected by the antibiotic presence, although all hairy roots showed the presence of the *npt-II *gene as determined by PCR (Additional file 1, Figure S1d). Furthermore, it has previously been shown that in soybean roots cultured at 22°C, DBI values of lipids increased with time and that other ratios (mol% 18:2/18:3 and RI = [%(18:2+18:3)]/%16:0) significantly declined between 7 and 26 days [46], in agreement with the PC2 component data (Figure 7). Similar trends were observed in the middle stages of the same soybean roots but not at the older stages.

## Conclusions

We have demonstrated that hairy roots of *E. acanthocarpum *are able to biosynthesize and accumulate a large and consistent range of polyunsaturated FAs, including the target GLA and SDA fatty acids, although the amounts of GLA, for example, were less than those described from seeds of the intact plant they are of significance.

Current studies are being undertaken to further optimize and establish a more productive n-3 PUFA *E. acanthocarpum *hairy root system, by varying other culture conditions *i.e. *increasing the osmotic pressure of the liquid nutrient medium and lowering the culture temperature (in an attempt to mimic the conditions of the deep sea environment), and changing the carbon source from sucrose to glucose. Furthermore, transgenic *E. acanthocarpum *hairy roots over-expressing a Δ6-desaturase gene are also being established in order to further manipulate the biosynthetic route aiming to boost SDA yields.

## Methods

### Plant Material

Seeds of *E. acanthocarpum*, donated by Jardín Botánico Viera y Clavijo (Gran Canaria, Spain), were surface sterilized by a brief immersion in 70% EtOH, followed by submersion in an aqueous solution of 5% (v/v) of commercial bleach for 25 min with gentle hand agitation. Finally, they were washed 5 times with sterile distilled water.

Surface sterilized seeds were then allowed to germinate *in vitro *on a solid B5 [47] medium, supplemented with 3% sucrose, 3-4 mg/L GA3 (gibberelic acid), and solidified with 0.7% agar, with the pH adjusted to 6.0 prior to autoclaving, contained in Petri dishes (90 mm diameter), and cultured in the dark until beginning of germination. Following germination, the plants were transferred to the same solid nutrient medium without the addition of GA3, contained in translucent glass jars covered with a lid (175 mL capacity, Sigma-Aldrich, MO, US), which were placed under light conditions (16 h photoperiod and irradiance of 35 mmol m^2^s^-1 ^supplied by cool-white fluorescent tubes) and a temperature of 25 ± 2°C to allow further plant growth.

*In vitro *germinated 50-60 day old plants were employed for guided infection with *Agrobacterium rhizogenes *strain LBA1334 harbouring a pBIN19-gus intron plasmid by repeatedly stabbing the internodal stem areas with a fine needle containing bacteria [48]. Infected plants were returned to the same culture vessel until hairy roots emerged. Hairy roots of 3-4 mm in length that developed after 25-30 days were aseptically excised from the infected stems, and transferred to a liquid medium as above but without agar, containing the antibiotic cefotaxime (100 mg/L), as well as 1% of the antioxidant polyvinylpyrrolidone (PVP) for several subcultures. Finally, actively growing bacterium-free hairy roots were cut into small segments and routinely cultured and refreshed in Erlenmeyer flasks (250 mL), containing 30 mL of sterile liquid B5 medium supplemented with 3% sucrose and 1% of PVP, sealed with a double layer of aluminium foil, and placed on an orbital shaker at 95 rpm in the dark at 25 ± 2°C.

For culture growth, fatty acid production and analysis, three hairy root cultures of the established two cell lines were investigated, i.e. hairy root line *HR E1.5 *grown in the quoted B5 liquid medium and conditions cultured for 35 days (sampled every 5 days); hairy root line *HR E1.16 *cultured for 50 days (sampled every 7 days), and hairy root line *HR E1.16 *cultured in the presence of kanamycin (30 mg/L) for 100 days (sampled every 14 days). In order to cover the entire growth period for each cell line, sampling times were different since the kinetic of growth differed due mainly to the cell line and the addition of kanamycin into the nutrient medium.

### Lipid extraction

Hairy roots cultures were separated from the liquid nutrient medium by vacuum filtration, after its pH was measured. The roots were then weighed and lyophilised at -80°C for 24 h using a freeze-dryer (Christ Alpha 2-4, Osterode, Germany). Freeze-dried samples were separately powdered using a mortar and pestle with liquid nitrogen. After homogenisation, total lipid of the samples was extracted following the method previously described [49,32].

### Transesterification of lipids

Total lipid aliquots (2 mg) were subjected to acid catalyzed transesterification by dissolving the sample in 1 mL toluene, employed to ensure that the neutral lipids got properly dissolved, plus 2 mL of a mixture of MeOH/1% H_2_SO_4_, and incubated in a capped glass test tube at 50°C for 16 h [50]. Prior to transmethylation, heneicosaenoic acid (21:0) was added to the lipid extracts as internal standard (2.5% of the total lipid analysed, 50 μg).

Transesterification was followed by the addition of 2 mL of an aqueous solution of K_2_CO_3 _(2% w/v) and 5 mL of hexane/ethyl ether (1:1, v/v), plus 0.01% butylated hydroxytoluene (BHT, w/v) followed by strong agitation. The mixture was centrifuged at 1500 rpm (239 g) at 4°C for 5 min. The upper phase was kept and the lower phase washed again with 5 mL of hexane/ethyl ether (1:1, v/v), the two upper phases were pooled together and evaporated under a stream of N_2_. Finally, the resulting fatty acid methyl esters (FAMEs) samples were dissolved in 100 mL hexane contained in sealed glass GC vials, kept at -20°C until required for analysis.

Isolation and purification of the FAMEs was conducted by preparative thin layer chromatography employing silica gel G-25 glass sheets (Macherey-Nagel, Germany), developed with a solvent system composed of hexane/diethyl ether/acetic acid 97.7% (90:10:1, by vol), and visualized after sublimation of iodine slightly heated. The FAMEs, which ran close to the solvent front, were scrapped off the glass sheet and extracted with 10 mL hexane/ethyl ether (1:1, v/v). Finally, the samples were dissolved in 0.5-1.0 mL hexane and kept under nitrogen in sealed glass vials at -20°C until analysis.

### Gas chromatography of FAMEs

Analysis and quantification of FAMEs was conducted by GC, employing a Shimadzu GC-14A apparatus (Shimadzu, Japan) equipped with a flame ionization detector (250°C), a Supelcowax™ 10 fused silica capillary column (30 m × 0.32 mm ID), and helium employed as carrier gas. Samples (0.6 mL) were injected into the system by an on-column auto-injector (Shimadzu AOC-17) at 50°C. For separation of compounds a temperature program of 180°C first 10 min, followed by an increase of 2.5°C/min to reach the final temperature of 215°C was employed.

FAMEs were identified according to their RT compared with standards of commercial FAMEs (linoleic acid methyl ester, methyl gamma-linolenate, methyl oleate, stearidonic acid methyl ester, and heneicosanoid acid), and a well-characterized fish oil mix. They were quantified according to the amount of 21:0 used as internal standard prior to transmethylation, and comparison with a calibration curve employing these standards.

### Statistical analysis

Results are presented as means ± SD (n = 3 for each sampling time, n = 21 for each kind of hairy root culture). The data were checked for normal distribution by the one-sample Kolmogorov-Smirnoff test, as well as for homogeneity of the variance with the Levene test, and when necessary, Bartlett test was also applied. When variance was not homogeneous, a Kruskal-Wallis and Games-Howel tests were conducted to assess statistical differences. The effects of culture conditions and sampling time of the studied parameters were firstly determined using one-way ANOVA-test (p < 0.05). The percentages and total amounts of FAs in the three different cultures were included as variables in a principal component analysis (PCA). Principal components were subsequently analysed by two-way ANOVA to study the combined effects of both, hairy root line and age of culture, as well as their interconnections. Statistical analyses were performed employing the SPSS software (versions 15.0 and 17.0, SPSS Inc, IL, USA).

## Competing interests

The authors declare that they have no competing interests.

## Authors' contributions

EC carry out the experiments, analyzed the experimental data and drafted the manuscript. RDG designed and supervised all statistical analyses of the data. RZ conceived the project and coordinated it, designed the study, revised the data and refined the manuscript. CR coordinated the project, revised the data and refined the manuscript. AGR contributed towards lab infrastructure and assisted in the performance of some analytical techniques. All authors have read and approved the final version of the manuscript.

## Supplementary Material

Additional file 1 **Tables (S1-S6) and Images S1a-f**. Tables S1-S5 show the different sampling points not included in the main text. In each table the total lipid content and general fatty acid profiles (%) of the two cell lines of *Echium acanthocarpum *hairy roots at different sampling points are presented. n-6 and n-3 Δ6-Desaturation Indexes were calculated as 18:3n-6/(18:3n-6+18:2n-6) and 18:4n-3/(18:4n-3+18:3n-3), respectively. DBI was calculated as [(% 18:1) +2*(% 18:2) +3*(% 18:3) +4*(18:4)]/100. Values are presented as the average of three replicates. Table S6 shows the results of two-way ANOVA analyses of the two principal components, PC1 and PC2, of the percentages of FAs. Images S1a-f illustrate images of the hairy root induction, the physical appearance of two hairy root lines, an agarose gel showing a PCR amplified kanamycin resistant gene (*npt-II*), as well as a *gus *assay photographs. Click here for file

Additional file 2 **Figures S1-S3**. Figures S1-S3 show plots of PC1 and PC2 factor scores categorized by type of culture and by sampling points. Click here for file
