# Supplementary material for: Echium acanthocarpum hairy root cultures, a suitable system for polyunsaturated fatty acid studies and production
Source: BMC Biotechnol. 2011 Apr 27;11:42. doi: 10.1186/1472-6750-11-42 (PMC3114721; doi:10.1186/1472-6750-11-42)
Supplement: Additional file 1 — Tables (S1-S6) and Images S1a-f. Tables S1-S5 show the different sampling points not included in the main text. In each table the total lipid content and general fatty acid profiles (%) of the two cell lines of Echium acanthocarpum hairy roots at different sampling points are presented. n-6 and n-3 Δ6-Desaturation Indexes were calculated as 18:3n-6/(18:3n-6+18:2n-6) and 18:4n-3/(18:4n-3+18:3n-3), respectively. DBI was calculated as [(% 18:1) +2*(% 18:2) +3*(% 18:3) +4*(18:4)]/100. Values are presented as the average of three replicates. Table S6 shows the results of two-way ANOVA analyses of the two principal components, PC1 and PC2, of the percentages of FAs. Images S1a-f illustrate images of the hairy root induction, the physical appearance of two hairy root lines, an agarose gel showing a PCR amplified kanamycin resistant gene (npt-II), as well as a gus assay photographs. [file 1472-6750-11-42-S1.PDF]

Sampling point 1	*HR E1.5*	*HR E1.16*	*HR E1.16 with kn*
Total Lipid content (mg/g DW)	***60.95 ± 6.10***	***43.01 ± 9.21***	***37.78 ± 9.74***
14:0	*0.83 ± 0.23*	*0.85 ± 0.11*	*0.46 ± 0.06*
16:0	*26.64 ± 1.03*	*24.00 ± 0.84*	*20.91 ± 0.59*
18:0	*3.78 ± 0.20*	*4.35 ± 0.01*	*4.22 ± 0.28*
18:1n-9	*6.29 ± 1.13*	*5.61 ± 0.53*	*6.99 ± 0.60*
18:1 n-7	*2.05 ± 0.17*	*1.24 ± 0.15*	*0.88 ± 0.02*
18:2n-6 (LA)	*33.57 ± 1.42*	*38.84 ± 0.46*	*39.42 ± 0.61*
18:3n-6 (GLA)	*10.46 ± 0.86*	*12.28 ± 0.15*	*9.18 ± 0.11*
18:3n-3 (ALA)	*5.13 ± 0.28*	*3.43 ± 0.09*	*6.00 ± 0.10*
18:4n-3 (SDA)	*1.33 ± 0.11*	*0.39 ± 0.12*	*1.06 ± 0.07*
20:0	*0.34 ± 0.01*	*0.44 ± 0.01*	*0.41 ± 0.02*
22:0	*2.11 ± 0.06*	*2.46 ± 0.14*	*2.71 ± 0.44*
24:0	*1.58 ± 0.12*	*2.04 ± 0.03*	*3.24 ± 0.72*
unknown	*4.88 ± 0.12*	*3.77 ± 1.22*	*3.58 ± 0.22*
Fatty acids (% of the total lipid content)	*15.13 ± 2.12*	*17.48 ± 7.47*	*17.19 ± 1.38*
GLA and SDA	*11.79 ± 0.97*	*13.37 ± 1.31*	*10.24 ± 0.18*
Saturated fatty acids	*35.28 ± 1.50*	*34.13 ± 0.92*	*31.95 ± 0.30*
Monoenefatty acids	*8.89 ± 0.71*	*7.42 ± 0.36*	*8.81 ± 0.62*
n-9	*6.44 ± 0.53*	*6.18 ± 0.52*	*7.76 ± 0.61*
n-6	*44.03 ± 2.18*	*51.12 ± 0.61*	*48.60 ± 0.60*
n-3	*6.45 ± 0.31*	*3.82 ± 0.03*	*7.06 ± 0.07*
n-3/n-6	*0.15 ± 0.01*	*0.09 ± 0.02*	*0.15 ± 0.00*
n-6 Δ6-Desaturation Index	*0.24 ± 0.01*	*0.28 ± 0.08*	*0.19 ± 0.00*
n-3 Δ6-Desaturation Index	*0.21 ± 0.01*	*0.10 ± 0.03*	*0.15 ± 0.01*
DBI	*1.29 ± 0.05*	*1.27 ± 0.13*	*1.37 ± 0.01*

Sampling point 2	*HR E1.5*	*HR E1.16*	*HR E1.16 with kn*
Total Lipid content (mg/g DW)	***40.74 ± 1.92***	***40.03 ± 2.07***	***38.20 ± 9.76***
14:0	*0.49 ± 0.12*	*0.47 ± 0.08*	*0.42 ± 0.04*
16:0	*26.84 ± 0.79*	*23.93 ± 0.09*	*21.28 ± 0.04*
18:0	*3.08 ± 0.16*	*4.10 ± 0.34*	*5.68 ± 0.29*
18:1n-9	*5.25 ± 0.73*	*5.05 ± 0.45*	*5.68 ± 0.25*
18:1 n-7	*1.76 ± 0.05*	*1.40 ± 0.42*	*0.94 ± 0.13*
18:2n-6 (LA)	*36.14 ± 1.15*	*41.41 ± 0.69*	*40.47 ± 0.93*
18:3n-6 (GLA)	*12.05 ± 0.41*	*11.55 ± 0.35*	*9.78 ± 0.79*
18:3n-3 (ALA)	*4.42 ± 0.22*	*3.59 ± 0.24*	*5.74 ± 0.30*
18:4n-3 (SDA)	*1.15 ± 0.10*	*0.42 ± 0.09*	*1.05 ± 0.03*
20:0	*0.41 ± 0.13*	*0.43 ± 0.22*	*0.41 ± 0.02*
22:0	*2.06 ± 0.12*	*2.39 ± 0.15*	*2.74 ± 0.32*
24:0	*1.56 ± 0.04*	*2.13 ± 0.10*	*3.47 ± 0.61*
unknown	*3.86 ± 0.62*	*2.62 ± 0.25*	*3.26 ± 0.10*
Fatty acids (% of the total lipid content)	*20.92 ± 2.53*	*30.96 ± 4.89*	*17.70 ± 5.00*
GLA and SDA	*13.2 ± 0.51*	*11.97 ± 0.44*	*10.83 ± 0.82*
Saturated fatty acids	*34.44 ± 0.50*	*33.46 ± 0.76*	*32.44 ± 1.30*
Monoene fatty acids	*7.93 ± 0.91*	*6.95 ± 0.54*	*7.55 ± 0.41*
n-9	*5.81 ± 0.83*	*5.54 ± 0.45*	*6.56 ± 0.29*
n-6	*48.20 ± 1.52*	*52.96 ± 0.65*	*49.43 ± 1.41*
n-3	*5.57 ± 0.26*	*4.01 ± 0.29*	*6.89 ± 0.28*
n-3/n-6	*0.12 ± 0.00*	*0.08 ± 0.00*	*0.14 ± 0.00*
n-6 Δ6-Desaturation Index	*0.25 ± 0.00*	*0.22 ± 0.01*	*0.19 ± 0.01*
n-3 Δ6-Desaturation Index	*0.21 ± 0.02*	*0.10 ± 0.02*	*0.15 ± 0.01*
DBI	*1.34 ± 0.05*	*1.37 ± 0.02*	*1.37 ± 0.04*

Tables S1 and S2. Total lipid content and general fatty acid profiles (%) of the two cell lines of *Echium acanthocarpum* hairy roots at sampling points 1 and 2 cultured in B5 nutrient liquid medium at 25°C. n-6 and n-3 Δ6-Desaturation Indexes were calculated as 18:3n-6/(18:3n-6+18:2n-6) and 18:4n-3/(18:4n-3+18:3n-3) respectively. DBI was calculated as [(% 18:1) +2*(% 18:2) +3*(% 18:3) +4*(18:4)]/100. Values are presented as the average of three replicates.

Sampling point 3	*HR E1.5*	*HR E1.16*	*HR E1.16 with kn*
Total Lipid content (mg/g DW)	***38.13 ± 5.35***	***43.32 ± 3.37***	***35.54 ± 8.32***
14:0	*0.39 ± 0.04*	*0.42 ± 0.03*	*0.49 ± 0.08*
16:0	*26.19 ± 0.61*	*24.86 ± 0.62*	*22.34 ± 1.30*
18:0	*2.98 ± 0.12*	*4.27 ± 0.34*	*4.30 ± 0.09*
18:1n-9	*6.38 ± 0.57*	*4.99 ± 0.28*	*4.38 ± 0.47*
18:1 n-7	*1.74 ± 0.07*	*1.58 ± 0.04*	*0.83 ± 0.03*
18:2n-6 (LA)	*36.10 ± 1.39*	*40.72 ± 0.65*	*39.99 ± 0.89*
18:3n-6 (GLA)	*13.21 ± 0.67*	*10.99 ± 0.36*	*9.55 ± 0.49*
18:3n-3 (ALA)	*4.11 ± 0.40*	*3.30 ± 0.05*	*5.79 ± 1.06*
18:4n-3 (SDA)	*1.12 ± 0.03*	*0.45 ± 0.17*	*1.01 ± 0.02*
20:0	*0.39 ± 0.03*	*0.33 ± 0.12*	*0.53 ± 0.04*
22:0	*2.39 ± 0.15*	*2.74 ± 0.20*	*3.60 ± 0.53*
24:0	*1.40 ± 0.10*	*2.60 ± 0.18*	*3.68 ± 0.68*
unknown	*2.66 ± 0.07*	*1.90 ± 0.43*	*3.60 ± 0.74*
Fatty acids (% of the total lipid content)	*19.50 ± 3.84*	*28.11 ± 4.24*	*15.68 ± 2.14*
GLA and SDA	*14.33 ± 0.70*	*11.44 ± 0.53*	*10.56 ± 0.51*
Saturated fatty acids	*33.73 ± 0.79*	*35.23 ± 1.23*	*34.95 ± 0.89*
Monoene fatty acids	*9.07 ± 0.59*	*7.40 ± 0.48*	*5.72 ± 0.34*
n-9	*7.00 ± 0.57*	*5.46 ± 0.28*	*4.80 ± 0.30*
n-6	*49.31 ± 1.00*	*51.71 ± 0.31*	*49.54 ± 0.93*
n-3	*5.23 ± 0.39*	*3.76 ± 0.16*	*6.80 ± 1.07*
n-3/n-6	*0.11 ± 0.01*	*0.07 ± 0.00*	*0.14 ± 0.02*
n-6 Δ6-Desaturation Index	*0.27 ± 0.02*	*0.21 ± 0.01*	*0.19 ± 0.01*
n-3 Δ6-Desaturation Index	*0.21 ± 0.02*	*0.12 ± 0.04*	*0.15 ± 0.02*
DBI	*1.38 ± 0.02*	*1.34 ± 0.01*	*1.36 ± 0.03*

Sampling point 6	*HR E1.5*	*HR E1.16*	*HR E1.16 with kn*
Total Lipid content (mg/g DW)	***31.38 ± 1.48***	***34.14 ± 6.81***	***30.88 ± 4.43***
14:0	*0.19 ± 0.01*	*0.20 ± 0.04*	*0.30 ± 0.20*
16:0	*25.39 ± 0.54*	*24.28 ± 0.79*	*24.71 ± 1.49*
18:0	*2.57 ± 0.12*	*3.71 ± 0.09*	*3.72 ± 0.18*
18:1n-9	*4.14 ± 0.54*	*5.19 ± 0.79*	*4.60 ± 1.33*
18:1 n-7	*2.30 ± 0.21*	*1.57 ± 0.16*	*1.28 ± 0.33*
18:2n-6 (LA)	*38.13 ± 0.52*	*43.54 ± 1.45*	*40.78 ± 4.00*
18:3n-6 (GLA)	*14.17 ± 0.85*	*10.19 ± 0.33*	*11.55 ± 1.48*
18:3n-3 (ALA)	*5.27 ± 0.21*	*3.61 ± 0.36*	*4.06 ± 0.60*
18:4n-3 (SDA)	*0.76 ± 0.21*	*0.32 ± 0.15*	*0.50 ± 0.27*
20:0	*0.19 ± 0.01*	*0.23 ± 0.03*	*0.32 ± 0.14*
22:0	*2.62 ± 0.22*	*2.81 ± 0.44*	*2.88 ± 0.39*
24:0	*1.75 ± 0.07*	*2.48 ± 0.48*	*2.65 ± 0.22*
unknown	*1.77 ± 0.01*	*1.33 ± 0.28*	*2.15 ± 1.28*
Fatty acids (% of the total lipid content)	*23.67 ± 2.51*	*27.76 ± 3.05*	*20.72 ± 8.72*
GLA and SDA	*14.93 ± 1.06*	*10.51 ± 0.48*	*12.05 ± 1.71*
Saturated fatty acids	*32.71 ± 0.52*	*33.71 ± 1.45*	*34.58 ± 2.15*
Monoene fatty acids	*7.20 ± 0.60*	*7.29 ± 0.76*	*6.39 ± 1.64*
n-9	*4.82 ± 0.46*	*5.72 ± 0.81*	*5.11 ± 1.39*
n-6	*52.29 ± 1.15*	*53.73 ± 1.47*	*52.32 ± 2.63*
n-3	*6.03 ± 0.33*	*3.93 ± 0.51*	*4.55 ± 0.86*
n-3/n-6	*0.12 ± 0.01*	*0.07 ± 0.01*	*0.09 ± 0.02*
n-6 Δ6-Desaturation Index	*0.27 ± 0.01*	*0.19 ± 0.01*	*0.22 ± 0.04*
n-3 Δ6-Desaturation Index	*0.13 ± 0.03*	*0.08 ± 0.03*	*0.10 ± 0.04*
DBI	*1.45 ± 0.02*	*1.37 ± 0.02*	*1.37± 0.03*

Tables S3 and S4. Total lipid content and general fatty acid profiles (%) of the two cell lines of *Echium acanthocarpum* hairy roots at sampling points 3 and 6 cultured in B5 nutrient liquid medium at 25°C. n-6 and n-3 Δ6-Desaturation Indexes were calculated as 18:3n-6/(18:3n-6+18:2n-6) and 18:4n-3/(18:4n-3+18:3n-3) respectively. DBI was calculated as [(% 18:1) +2*(% 18:2) +3*(% 18:3) +4*(18:4)]/100. Values are presented as the average of three replicates

Sampling point 7	*HR E1.5*	*HR E1.16*	*HR E1.16 with kn*
Total Lipid content (mg/g DW)	***31.59 ± 3.45***	***32.31 ± 3.41***	***45.29 ± 3.40***
14:0	*0.15 ± 0.05*	*0.58 ± 0.09*	*0.41 ± 0.21*
16:0	*26.27 ± 1.33*	*24.87 ± 0.66*	*24.76 ± 0.66*
18:0	*2.56± 0.03*	*3.92 ± 0.08*	*3.81 ± 0.29*
18:1n-9	*4.40 ± 0.57*	*5.36 ± 0.32*	*4.00 ± 0.64*
18:1 n-7	*2.46 ± 0.22*	*1.23 ± 0.59*	*0.86 ± 0.06*
18:2n-6 (LA)	*38.75 ± 0.47*	*42.21 ± 1.11*	*38.41 ± 0.10*
18:3n-6 (GLA)	*12.89 ± 1.63*	*12.06 ± 0.43*	*12.02 ± 0.26*
18:3n-3 (ALA)	*4.69 ± 0.76*	*3.48 ± 0.33*	*4.67 ± 0.26*
18:4n-3 (SDA)	*0.47 ± 0.25*	*0.57 ± 0.09*	*0.82 ± 0.08*
20:0	*0.22 ± 0.05*	*0.41 ± 0.23*	*0.47 ± 0.03*
22:0	*2.81 ± 0.10*	*1.98 ± 0.28*	*2.61 ± 0.50*
24:0	*1.70 ± 0.17*	*1.00 ± 0.39*	*1.52 ± 0.59*
unknown	*2.01 ± 0.17*	*2.08 ± 0.93*	*4.28 ± 1.76*
Fatty acids (% of the total lipid content)	*23.45 ± 2.35*	*25.98 ± 0.24*	*17.83 ± 4.13*
GLA and SDA	*13.36 ± 1.88*	*12.63 ± 0.82*	*12.84 ± 0.34*
Saturated fatty acids	*33.71 ± 1.23*	*32.76 ± 0.05*	*33.57 ± 1.09*
Monoene fatty acids	*7.48 ± 0.77*	*6.84 ±0.01*	*5.87 ± 0.68*
n-9	*5.02 ± 0.56*	*5.56 ± 0.52*	*4.46 ± 0.71*
n-6	*51.64 ± 1.17*	*54.27 ± 1.35*	*50.43 ± 0.18*
n-3	*5.16 ± 1.00*	*4.05 ± 0.76*	*5.49 ± 0.33*
n-3/n-6	*0.10 ± 0.02*	*0.08 ± 0.02*	*0.11 ± 0.01*
n-6 Δ6-Desaturation Index	*0.25 ± 0.03*	*0.22 ± 0.00*	*0.24 ± 0.00*
n-3 Δ6-Desaturation Index	*0.09 ± 0.04*	*0.13 ± 0.06*	*0.15 ± 0.01*
DBI	*1.40 ± 0.06*	*1.40 ± 0.00*	*1.36 ± 0.01*

Table S5. Total lipid content and general fatty acid profiles (%) of the two cell lines of *Echium acanthocarpum* hairy roots at sampling point 7 cultured in B5 nutrient liquid medium at 25°C. n-6 and n-3 Δ6-Desaturation Indexes were calculated as 18:3n-6/(18:3n-6+18:2n-6) and 18:4n-3/(18:4n-3+18:3n-3), respectively. DBI was calculated as [(% 18:1) +2*(% 18:2) +3*(% 18:3) +4*(18:4)]/100. Values are presented as the average of three replicates.

Two-Way ANOVA	*Culture*		*Sampling time*		*Interaction Culture-Sampling time*	
	F-valor	*Sign.*	*F-valor*	*Sign.*	*F-valor*	*Sign.*
CP1	*499.409*	*0.000*	*37.194*	*0.000*	*11.939*	*0.000*
CP2	*108.110*	*0.000*	*9.667*	*0.000*	*3.382*	*0.002*

Table S6. Two-way ANOVA of the two principal components, PC1 and PC2, of the percentages of FAs. Sign.: Signification (p<0.05).

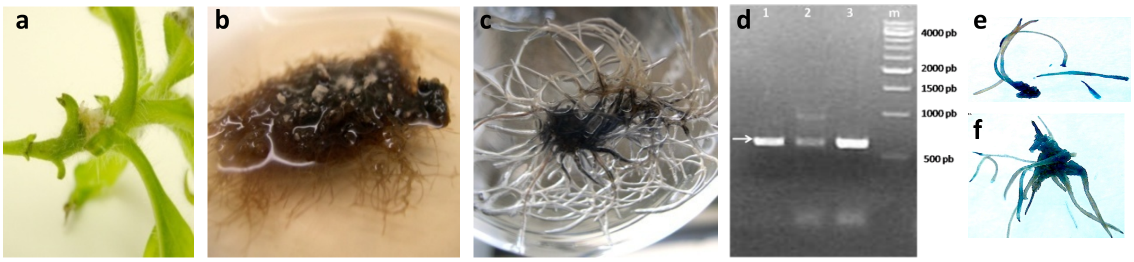

Images S1a-f. a. Induction of hairy roots in a plant stem. **b-c**. Physical appearance of *HR E1.5* and *HRE1.16* hairy root cultures respectively. **d.** Agarose gel showing a PCR amplified product of kanamycin resistant gene (*npt-II*) detection. The arrow marks the expected 732 pb product in the three lanes. Lane 1= PCR product from *Atropa baetica* hairy root (*H6H*) containing *npt-II* gene (positive control); Lane 2= PCR product from *HR E1.5* hairy root line. The approximately 1000 pb PCR product corresponded to an unspecific amplification reaction. Lane 3= PCR product from *HR E1.16* hairy root line. m= molecular weight marker. The different intensity of PCR product (lane 2 *vs.* lane 3) could be explained by a *npt-II* gene minor copy number in *HR E1.5* hairy root DNA. **e-f.** Images of a *gus* assay of *HRE1.16* hairy root line.
