# Supplementary material for: Echium acanthocarpum hairy root cultures, a suitable system for polyunsaturated fatty acid studies and production
Source: BMC Biotechnol. 2011 Apr 27;11:42. doi: 10.1186/1472-6750-11-42 (PMC3114721; doi:10.1186/1472-6750-11-42)
Supplement: Additional file 2 — Figures S1-S3. Figures S1-S3 show plots of PC1 and PC2 factor scores categorized by type of culture and by sampling points. [file 1472-6750-11-42-S2.PDF]

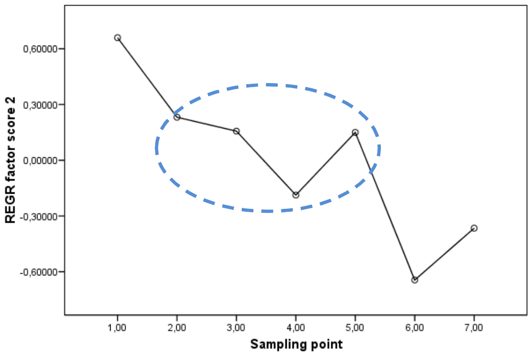

Figure 1S. Plot of PC2 factor scores categorized by sampling points.

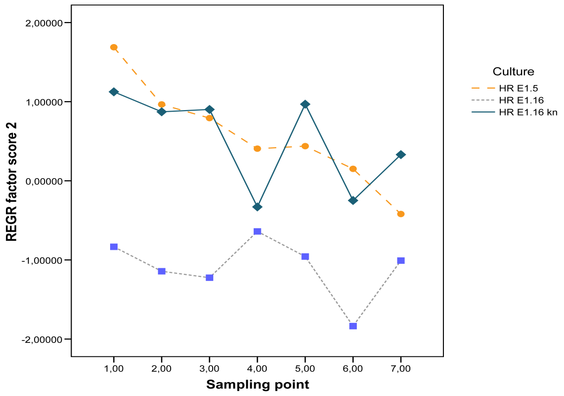

Figure 2S. Plot of PC2 factor scores categorized by type of culture and by sampling points.

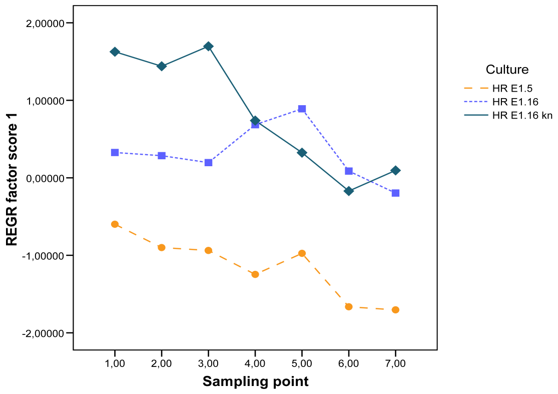

Figure 3S. Plot of PC1 factor scores categorized by type of culture and by sampling points.
